# Impact of Serum Prolactin and Testosterone Levels on the Clinical Parameters of Dry Eye in Pregnant Women

**DOI:** 10.1155/2020/1491602

**Published:** 2020-08-30

**Authors:** Samuel Kyei, Richard K. Dadzie Ephraim, Stephen Animful, Madison Adanusa, Stephen Karim Ali-Baya, Belinda Akorsah, Mabel Antwiwaa Sekyere, Kofi Asiedu

**Affiliations:** ^1^Department of Optometry and Vision Science, College of Health and Allied Sciences, University of Cape Coast, Cape Coast, Ghana; ^2^Department of Medical Laboratory Science, School of Allied Health Sciences, University of Cape Coast, Cape Coast, Ghana; ^3^Division of Family Medicine, Directorate of University Health Services, University of Cape Coast, Cape Coast, Ghana; ^4^Eye Clinic, Cosmopolitan Medical Center, North-Dzorwulu, Accra, Ghana

## Abstract

**Purpose:**

To explore the relationship between serum prolactin, testosterone level, and tear film parameters. The potential impact of these hormones on the health of the ocular surface in pregnant women was evaluated.

**Methods:**

This was a hospital-based cross-sectional study in which ocular symptoms (Ocular Surface Disease Index (OSDI)), tear function (fluorescein tear breakup time, Schirmer's test 1), corneal and conjunctival staining, meibomian gland (MG) expressibility, and quality of secretion were measured. Lid margin findings including lid notching, thickness, and lid margin telangiectasia were also recorded. Venous blood was collected and the serum concentrations of prolactin and testosterone were determined using ELISA kits. Correlation and multiple linear regression analyses were used to examine predictors of dry eye symptoms and signs.

**Results:**

A total of 160 pregnant women participated in the study and their mean age was 30.1 ± 4.6 years (range 18–42 years). The correlation analysis indicated that testosterone concentration did not significantly correlate with any of the clinical parameters hence it was not included in the linear regression analysis. However, prolactin serum concentration correlated significantly with Schirmer's test 1. Multiple linear regression was done to predict participants' Schirmer's test 1 score based upon their ocular surface staining score (oxford grading scale), meibomian gland expressibility, meibomian secretion quality, and serum concentration of prolactin. Only predictors that significantly correlated with Schirmer's test 1 in the correlation analysis were included in the linear regression analysis. A significant regression equation was obtained (*F* (2, 157) = 5.119, *p*=0.007) with an *R* square of 0.05. Multiple linear regression analysis revealed that concentration of prolactin (ß coefficient = 0.032, *p*=0.044) and meibomian gland expressibility scores (ß coefficient = 2.14, *p*=0.016) were associated with Schirmer's test 1 scores when adjusted for duration of pregnancy.

**Conclusion:**

The study showed that increased serum prolactin levels have a weak but positive impact on Schirmer's test 1 scores whilst serum testosterone level has no association with the clinical parameters of dry eye in pregnant women.

## 1. Introduction

Dry eye disease is one of the commonest conditions encountered by optometrists and ophthalmologists in clinical practice [1]. It has been demonstrated in several studies that dry eye has a huge impact on the quality of life and psychological states of its sufferers [1–3]. Dry eye disease is a multifactorial disease characterized by loss of homeostasis of the tear film and frequently accompanied by ocular symptoms [4]. One of the major explored risk factors for dry eye disease has been the impact of hormones especially sex hormones and usage of oral contraceptives [1, 5, 6].

Many studies have reported a higher prevalence of dry eye disease in women compared to men [1]. This has further increased research interest on the impact of hormones in dry eye disease, particularly the influence of sex hormones, as there is the plausibility of hormones being responsible for the difference in prevalence between males and females.

In intracrinology, it has been demonstrated that sex steroid receptor mRNAs are present in the meibomian glands, lacrimal glands, and other human ocular surface tissues [6, 7]. Estrogen, androgen, and progesterone receptor proteins are located in the epithelial cell nuclei of human meibomian glands, cornea, lacrimal glands, forniceal and bulbar conjunctiva, which signify translation of sex steroid receptor mRNAs [6, 8] Lacrimal and meibomian glands have also been demonstrated to have receptors for peptide hormones such as prolactin [6, 9, 10].

Even though there is evidence for the local synthesis of hormones in ocular surface tissues [11], the systemic circulation of these hormones may also have a significant impact on ocular tissues. To truly know the impact of serum prolactin levels on tear film parameters, it is imperative to study its impact in pregnant women in whom serum prolactin levels are at an all-time high level compared to nonpregnant women [12, 13]. In males, prolactin has been shown to have a gonadotropic effect that makes the testes more sensitive to luteinizing hormone [12]. Thus, it indirectly enhances the secretion of testosterone [12]. Ironically, testosterone has been shown to downregulate the prolactin receptor gene in the lacrimal gland and other tissues [6, 13]. Testosterone levels are increased in pregnant women compared to nonpregnant women [14] and its known effect on prolactin receptor downregulation warrant a study [6, 15] involving both hormones to ascertain their impact on tear film parameters.

According to the DEWS II report (Sex, Gender and Hormone Subcommittee Report), the species-independent role of prolactin as a hormone on tears film dynamics and the lacrimal apparatus is unknown [6]. This study sought to investigate the relationship between serum prolactin, testosterone levels, and tear film parameters and their potential impact on ocular surface health in pregnant women.

## 2. Methods

This was a hospital-based cross-sectional study. Subjects for the study were consecutive pregnant women visiting the University of Cape Coast Hospital. All pregnant women, irrespective of age, gestational period, parity, etc., who visited the antenatal clinic of the University of Cape Coast Hospital within the study period and met the inclusion criteria were recruited. Subjects were included in the study if they did not have any of the following: lid margin abnormalities (Chalazion, Stye, etc.), ocular surface abnormalities (Pterygium, Pinguecula, Band keratopathy, Salzmann nodular degeneration, Spheroidal degeneration, etc.), systemic disease (Diabetes, Hypertension, Sjogren syndrome, etc.), history of contact lens wear, history of ocular surgeries, and usage of local or systemic medications that can affect the tear function.

Written informed consent was obtained from each participant. All clinical protocols and procedures for the study were conducted per the tenets of the Declaration of Helsinki for the use of human subjects in research. The study was approved by the institutional review board of the University of Cape Coast with ethical clearance number UCCIRB/CHAS/2017/49. Similarly, permission was sort from the University of Cape Coast Health Directorate before the commencement of the study. There were an attending midwife and obstetrician-gynecologist present, who attended to any health issues outside the scope of the eye care team during the period of data collection. The rationale, procedures, risks, and benefits of the study were comprehensively explained to all participants after whom a written consent was obtained. The participants were assured of confidentiality and safety at all times. They were also reminded of the voluntary nature of the study, which grants them the right to withdraw at any point.

### 2.1. Clinical Assessments

All clinical assessments were done by a well-trained optometrist on the team. Participants completed the Ocular Surface Disease Index (OSDI) questionnaire before commencing the clinical examination. The clinician was not made aware of the symptom score on the symptom questionnaire. The clinical assessment performed included fluorescein tear breakup time, conjunctival staining with Lissamine green, corneal fluorescein staining, Schirmer test without anesthesia, meibomian gland expressibility, and lid margin assessments with the slit lamp biomicroscope. All clinical assessments were made for each eye.

### 2.2. Symptom Assessments

The Ocular Surface Disease Index (developed by the Outcome Research group at Allergan (Irvine, CA)) is a 12-item questionnaire created to allow easy evaluation of the symptoms of dry eye [16, 17]. The total ocular surface disease index score was calculated for each subject based on the formula: the sum of scores of all items answered multiplied by 25 divided by the total number of items answered [17].

### 2.3. Schirmer 1 Test

The folded end of a 35 mm long and 5 mm wide precalibrated (mm) Whitman no 41 (Optitech eye care, Allahabad, India) paper was gently inserted into the lateral 1/3rd of the lower fornix without touching the cornea. The extent of the wetting (mm) after 5 minutes using a stopwatch was recorded as the Schirmer 1 test score [18].

### 2.4. Tear Breakup Time (TBUT)

Without any anesthesia, dry fluorescein impregnated paper strip (Optitech eye care, Allahabad, India) was wetted with normal saline. The strip was applied to the temporal conjunctiva with the patient looking nasally after it has been fully wetted. The cornea was viewed with a slit lamp using cobalt blue filtered light with a yellow barrier filter (Wratten number 12 filter). The patient was instructed to blink twice and then stare straight ahead without blinking. The time from the last blink to the first occurrence of a dry spot or dark spot was measured three times with the help of a stopwatch. The mean of three measurements was recorded as the tear breakup time [19].

### 2.5. Conjunctival and Corneal Staining

Immediately, after the tear breakup time was measured, corneal staining was graded. Lissamine green impregnated paper strip (Optitech eye care, Allahabad, India) was also wetted with normal saline and applied on the temporal conjunctiva immediately after the corneal staining has been graded. One to two minutes' time interval was allowed after applying the Lissamine green dye and grading of the conjunctiva was done. The conjunctival and corneal staining was graded based on the Oxford grading scale ranging from 0 to 15 [20].

### 2.6. Meibomian Gland Expressibility and Meibum Quality

Moderate digital pressure was applied to the tarsus of the lower eyelid to express the central eight meibomian glands. The number of glands expressed, the degree of ease, and the clarity of the meibum secretion was noted using the slit lamp biomicroscope. Gland expressibility was graded as follows: 0 = all glands producing lipids (normal), 1 = three to four producing lipids, 2 = one to two producing lipids, 3 = no glands expressible [19]. Meibum secretions were similarly graded as follows: 0 = clear lipids easily expressed (normal), 1 = cloudy lipids expressed with minimal pressure, 2 = cloudy lipids with particles, and 3 = inspissated lipids (gel-like). The highest score for any of the expressed gland was taken as the score [19].

### 2.7. Lid Margin Assessments

Lid margin telangiectasia was graded on a scale from 0 to 3 : 0 = no lid margin redness and no telangiectasia crossing meibomian gland orifices, 1 = lid margin redness and no telangiectasia crossing meibomian gland orifices, 2 = telangiectasia crossing meibomian gland orifices with a distribution of less than half of the full length of the lid, and 3 = telangiectasia crossing meibomian gland orifices with a distribution of half or more of the full length of the lid [21]. Lid margin thickness: 0 = no lid margin thickening, 1 = lid margin thickening with or without focal rounding, and 2 = lid margin thickening with diffuse or complete rounding [21]. Lid margin notching: 0 = no notching is observed, 1 = shallow dimpling of the lid margin, and 2 = deep dimpling of the lid margin [21].

### 2.8. Assessment of Serum Testosterone and Prolactin

The assessment was carried out by a registered medical laboratory practitioner. At the sample collection point, 5 mL of venous blood was aseptically drawn from each participant using a 5 mL disposable needle and syringe after disinfecting the selected venipuncture site with 70% ethanol. The blood samples were dispensed into the gel separator tubes (yellow-covered tubes, Becton Dickson (BD) company, Franklin Lakes, New Jersey, USA), and the fluoride tubes (Grey-covered tubes) and labeled appropriately with corresponding participant's identification number and date of sample collection. The fluoride tubes are inversed for about 10 times to ensure complete mixture with anticoagulant (Sodium fluoride). The samples were kept at 25°C for 20 minutes to allow clotting and clot retraction. The samples were centrifuged 10 minutes at 3000 rpm with Sure-Sep II, silicon-based serum-plasma separator centrifuge (Organon Teknika Corporation, North Carolina, USA). Serum fractions were removed using Pasteur pipette, transferred into Eppendorf tubes, and labeled appropriately and stored at −20°C until needed.

All reagents were brought to room temperature (18–22°C) before use. Samples that were stored at −20°C were brought out and thawed at room temperature (22°C). The thawed samples were vortexed (REAX top, Made in Germany) to ensure homogeneity in the composition of serum.

### 2.9. Prolactin Assay

Serum prolactin (PRL) was determined using a commercially acquired kit (BioVendor–Laboratorní Medicína a.s., Czech Republic, Cat. No.: RCD023R). A volume of 25 *μ*L of each calibrator, control, and serum was pipetted into the microwells and labeled correspondingly. The wells were incubated on a plate shaker at 200 rpm for an hour at room temperature. The wells were washed 3 times with 300 *μ*L of diluted wash buffer per well and the plate was tapped firmly against absorbent paper to ensure that it is dry. A 150 *μ*L of TMB substrate was pipetted into each well at 2 minutes time intervals and further incubated for 10–15 minutes at room temperature or until calibrator F attains dark blue colour for desired OD. After 50 *μ*L of stopping, solution has been pipetted into each 96-well microtiter as per the manufacturer's instruction. It was read at 405 nm with a URIT-660 microplate reader (URIT Medical Electronic Co., Ltd, Guangxi, China). Each determination was done in duplicate.

### 2.10. Testosterone Assay

Serum testosterone was determined using a commercially acquired kit (BioVendor–Laboratorní Medicína a.s., Czech Republic, Cat. no.: RCD027R). A volume of 25 *μ*L of each calibrator, control, and serum was pipetted into the microwells and labeled correspondingly. A 100 *μ*l of the conjugate working solution was pipetted into each well and shaken gently for 10 seconds. The plate was then incubated at 37°C for 1 hour. The wells were washed 3 times with 350 *μ*L of diluted wash buffer per well and the plate was tapped firmly against absorbent paper to ensure that it is dry. A 150 *μ*L of TMB substrate was pipetted into each well at 2-minute time intervals and further incubated for 10–15 minutes at 37°C. After 50 *μ*L of stopping, the solution has been pipetted into each 96-well microtiter as per the manufacturer's instruction. It was read at 405 nm with a URIT-660 microplate reader (URIT Medical Electronic Co., Ltd, Guangxi, China) within 20 minutes. Each determination was done in duplicate.

### 2.11. Data Analysis

In all analyses, the result of the worst eye was used [22]. All statistical analyses were performed using SPSS version 21.0 (SPSS Inc., Chicago, IL) statistical package except for the adjusted *R*^2,^ which was manually calculated using Stein's formula. General linear regression analysis was used to develop models to examine predictors of dry eye symptoms and signs. In brief, Spearman's and Pearson's bivariate correlations were used to examine associations between the outcome variables (symptoms and signs of dry eye) and other independent variables. Those associated with the outcome variable at a level of *p* < 0.05 were entered into a general linear model (stepwise regression). Models were examined for outliers using the standardized residuals and influencers were examined using the Cook's distance and leverage to ensure the model was a good representation of the sampled data. Standardized residuals greater than ±3.29 were considered statistically significant [23]. Cook's distances greater than 1 were considered statistically significant and leverage values greater than twice the average value were considered statistically significant [23, 24]. To determine the generalizability of the regression model multicollinearity, homoscedasticity, lack of autocorrelation was determined. Multicollinearity was tested using the variance inflation factor (VIF) and tolerance. VIF with a value greater than 10 and tolerance <0.2 indicates serious multicollinearity [23, 25, 26]. A plot of the *standardized residuals* (*Y*-axis) against the *standardized predicted values* (*X*-axis) was done because this plot is useful in determining whether the assumptions of homoscedasticity and random errors were held. The assumption of lack of autocorrelation or independent errors was tested with the Durbin–Watson test. Durbin–Watson test close to 2 (1.5–2.0) was considered adequate [23]. Cross-validation of the model was determined by manually calculating adjusted *R*^2^ using Stein's formula to determine if there is any shrinkage or loss of predictive power [23].

## 3. Results

The mean age of the pregnant women who participated in the study was 30.1 ± 4.6 years (range 18–42 years). A total of 160 pregnant women were included in the analysis. Group mean results for measurements of serum hormones, ocular symptoms, and signs are presented in Table 1.

**Figure 1:**
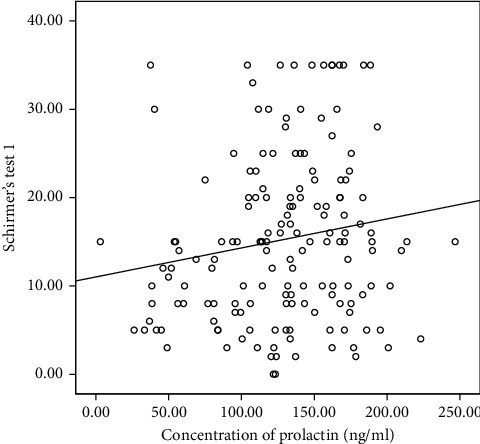
Correlation between prolactin and Schirmer's test score.

**Figure 2:**
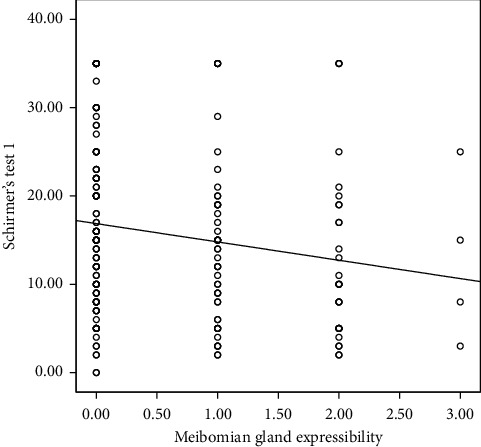
Correlation between Schirmers test 1 and meibomian gland expressibility.

**Figure 3:**
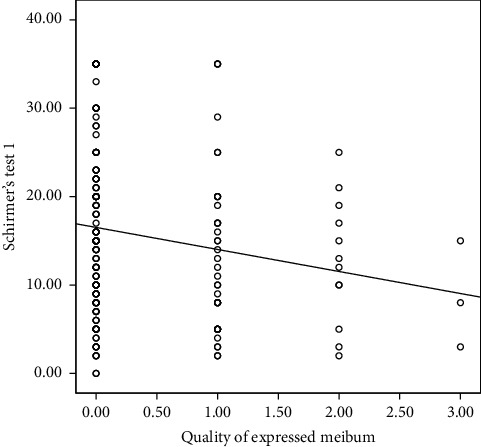
Correlation between Schirmer's test 1 and the quality of expressed meibum.

**Figure 4:**
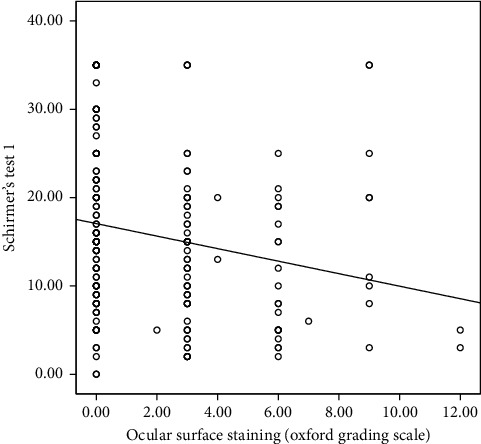
Correlation between Schirmer's test 1 and ocular surface staining (oxford grading scale).

The correlation analysis indicated that testosterone concentration did not significantly correlate with any of the clinical parameters; hence, it was excluded from the linear regression analysis. However, serum prolactin concentration correlated significantly with Schirmer's test 1. This is shown in Table 2. Multiple linear regression was done to predict participants' Schirmer's test 1 score based upon their ocular surface staining score (oxford grading scale), meibomian gland expressibility, meibomian secretion quality, and serum concentration of prolactin. Only predictors that significantly correlated with Schirmer's test 1 in the correlation analysis were included in the linear regression analysis. This is illustrated with the correlation graphs in Figures 1–4. Preliminary analyses were performed to ensure there was no violation of the assumptions of normality. A significant regression equation was (*F* (2, 157) = 5.119, *p*=0.007) with an *R* square of 0.05. Multiple linear regression analysis revealed that concentrations of prolactin (ß coefficient = 0.032, *p*=0.044) and meibomian gland expressibility scores (ß coefficient = 2.14, *p*=0.016) were associated with Schirmer's test 1 scores when adjusted for duration of pregnancy. This implies that participants, Schirmer's test 1 scores decreased by 2.1 mm for every 1 unit increase in meibomian gland expressibility score and every 1 ng/ml increase in prolactin concentration resulted in a 0.03 mm increase in Schirmer's test 1 score. Both serum prolactin concentration and meibomian gland expressibility scores were significant predictors. Among the clinical tests, only the Schirmer's test 1 had a statistically significant correlation with prolactin concentration. Hence, all clinical parameters with a statistically significant correlation coefficient with Schirmer's test 1 were entered into the multiple linear regression model. The standardized residuals of the model lay in-between −1.8–2.4 with a mean of 0 ± 1.00 indicating the lack of outliers. The average Cook's distance and leverage values were 0.06 ± 0.01 and 0.013 ± 0.012, respectively. No Cook's distance was greater than one and the leverage values were within less than twice the average indicating the absence of influencers in the model. The values of VIF of the predictors were between 1.0 and 1.634 less than 10 and an average tolerance of 0.722, indicating absence serious multicollinearity that may be biasing the model. The graph of the *standardized residuals* (*Y*-axis) against the *standardized predicted values* (*X*-axis) showed a random array of dot evenly dispersed around 0 indicating the gross absence of heteroscedasticity. The Durbin–Watson test was 1.86 indicating the assumption of the absence of autocorrelation was tenable. The adjusted *R*^2^ was 0.03 using Stein's formula which is similar to *R*^2^ = 0.05 indicating the cross-validity of the model. The summary of the multiple linear regression analysis is illustrated in Table 3.

## 4. Discussion

There was a statistically significant but weak positive association between prolactin and testosterone levels in this cohort of pregnant women which is consistent with the well-known fact that hyperprolactinemia is associated with elevated free testosterone levels in women [27, 28]. In pregnant women, the prolactin level is high; hence this finding may not be surprising. Furthermore, the correlation among the clinical signs and symptoms in this cohort of pregnant women was poor which is consistent with several studies in different populations [29, 30].

The study explored the impact of serum prolactin and testosterone levels on the clinical parameters of dry eye in pregnant women. Overall, the serum testosterone levels were not significantly associated with the signs and symptoms of dry eye in this cohort of pregnant women. First of all, it is known in animal and human studies that androgens including testosterone impact on sebaceous glands [31, 32]. Hence, it was expected that, as the meibomian glands are modified, sebaceous glands, physiology should have been impacted by testosterone. Surprisingly, this was not the case and several plausible reasons may account for this conundrum. It is possible that due to the increase in estrogen levels during pregnancy, it may competitively bind to androgen receptors on the ocular surface, thereby nullifying the effect of testosterone, especially on the meibomian glands [30]. Furthermore, higher estrogen levels may reduce the conversion of testosterone to its more potent form of dihydrotestosterone [31]. Again animal studies in hypophysectomized rats showed that a high level of prolactin (as observed in pregnant women) impairs dihydrotestosterone ability to support sodium pump expression in the lacrimal glands [33]. Besides, the study did not explore the local synthesis of testosterone by the ocular surface cells; hence it may be possible that the main source of testosterone might be its local synthesis by the ocular surface cells. Our findings on testosterone were consistent with Golebiowski et al. [31] who also found a lack of association between other androgens and the signs and symptoms of dry eye in postmenopausal women.

On the other hand, prolactin did not correlate with the majority of the clinical parameters of dry eye disease; however, there was a weak but statistically significant association between the serum prolactin levels and Schirmer test 1 scores. We utilized stepwise multiple linear regression analysis which is good at excluding shared variance among predictors but only the inherent variance; a predictor contributes to the outcome variable. The linear regression analysis showed that serum prolactin levels contributed weakly but significantly in the variance of the Schirmer test 1 scores. This may have some clinical significance because ocular surface aqueous production is multifactorial, including but not limited to, neuronal, hormonal, and external factors [34]. Arguably, prolactin may have a role to play in tear production. Studies have shown that prolactin receptors are expressed in the lacrimal gland acinar epithelial cells and the lacrimal glands actively secrete prolactin into tears [6]. Our findings are in sharp contrast with an earlier study [10] that showed a negative correlation between serum prolactin levels and tear function. There could be several reasons for this difference in findings. These may include differences in the study populations, study design, and ELISA kits used in either study. Our findings were in pregnant women as opposed to those of the other study which was in nonpregnant women [10].

To ensure that our sample regression model would not differ significantly from the population model characteristics, we cross-validated our regression model using Stein's formula which demonstrated minimal shrinkage of the coefficient of determination. This implies if a different sample of pregnant women was drawn from this same population, the probability of a similar finding is very high.

The current study only explored serum prolactin levels but the local synthesis of prolactin by the main lacrimal glands and accessory lacrimal glands was not studied. One may argue that prolactin may even have a more significant role in tear production if the local synthesis is fully explored in future intracrinology studies. Future studies should look at the impact of the local synthesis of these hormones on ocular surface parameters. There are minimal limitations with common laboratory techniques such as ELISA kits in the measurement of serum hormones; however, the application of newer techniques, such as mass spectroscopy, may improve assay sensitivity and specificity. Furthermore, serum hormone levels and Schirmer's test results may be influenced by other variables not considered in this study such as diet, smoking status, and health parameters.

In conclusion, the study showed increased serum prolactin levels have a weak but positive impact on Schirmer's test 1 scores but serum testosterone levels have no association with the clinical parameters of dry eye in pregnant women.

## Figures and Tables

**Table 1:** Group mean measurements for serum hormones, dry eye symptoms, and ocular surface signs.

Parameter (units)	Mean ± SD	Range

Tear breakup time (seconds)	(6.1 ± 3.1) s	2 s–15 s
Ocular surface staining	2.1 ± 2.7	0–12
Schirmer test 1 (mm)	15.2 ± 9.4	0–35
OSDI score	13.4 ± 16.1	0–87.5
Meibomian gland expressibility	0.6 ± 0.8	0–3
Quality of expressed meibum	0.4 ± 0.7	0–3
Lid margin thickness	0.3 ± 0.4	0–3
Lid margin telangiectasia	0.1 ± 0.4	0–3
Lid margin notching	0.1 ± 0.4	0–3
Prolactin (ng/mL)	128.6 ± 45.8	3.12–246.8
Testosterone (ng/mL)	69 ± 15.1	35–100.7
Age (years)	30 ± 4.7	18–42

**Table 2:** Correlation between serum hormones concentration and clinical parameters.

Parameter (unit)	SC	TBUT	OSS	MGE	MQ	LMT	LMN	TC	PC	OSDI

TC (ng/mL)	*R* = 0.09 *p*=0.24	*R* = 0.01 *p*=0.93	*R* = −0.02 *p*=0.82	*R* = −0.11 *p*=0.18	*R* = −0.01 *p*=0.95	*R* = 0.11 *p*=0.17	*R* = 0.08 *p*=0.92	1.0	*R* = 0.16 *p*=0.04	*R* = 0.01 *p*=0.9

PC (ng/mL)	*R* = 0.16 *p*=0.03	*R* = −0.06 *p*=0.48	*R* = −0.15 *p*=0.06	*r* = −0.04 *p*=0.64	*R* = −0.07 *p*=0.64	*R* = 0.11 *p*=0.17	*R* = 0.01 *p*=0.91	*R* = 0.16 *p*=0.04	1.00	*R* = 0.15 *p*=0.051

SC (mm)	1.00	*R* = 0.08 *p*=0.26	*R* = −0.24 *p* < 0.01	*R* = −0.2 *p*=0.005	*R* = −0.17 *p*=0.014	*R* = −0.06 *p*=0.41	*R* = −0.12 *p*=0.09	*R* = 0.09 *p*=0.24	*R* = 0.16 *p*=0.03	*R* = 0.09 *p*=0.91

MGE: meibomian gland expressibility; MQ: quality of secretion score; PC: prolactin concentration; TC: Testosterone concentration; OSS: ocular surface staining; LMT: lid margin thickness; LMN: lid margin notching; OSDI: ocular surface disease index; TBUT: tear breakup time; SC: Schirmer's test 1 score.

**Table 3:** Multiple linear regression analysis for predictors of Schirmer's test 1.

Schirmer's test 1	Unstandardized beta	Standard error	Standardized beta	Sig

Constant (model 1)	16.5	0.91		<0.001
Meibomian gland expressibility	−2.1	0.88	−0.19	0.016
Constant (model 2)	12.4	2.4		<0.001
Meibomian gland expressibility	−2.1	0.88	−0.19	0.016
Prolactin	0.03	0.016	0.16	0.044

Variables excluded by stepwise regression analysis, ocular surface staining, and quality of expressed meibum.

## Data Availability

The data for this paper are available upon reasonable request from the corresponding author.

## References

[1] Stapleton F., Alves M., Bunya V. Y. (2017). TFOS DEWS II epidemiology report. *The Ocular Surface*.

[2] Kim K. W., Han S. B., Han E. R. (2011). Association between depression and dry eye disease in an elderly population. *Investigative Ophthalmology & Visual Science*.

[3] Asiedu K., Dzasimatu S. K., Kyei S. (2018). Impact of dry eye on psychosomatic symptoms and quality of life in a healthy youthful clinical sample. *Eye & Contact Lens*.

[4] Craig J. P., Nichols K. K., Akpek E. K. (2017). TFOS DEWS II definition and classification report. *The Ocular Surface*.

[5] Asiedu K., Kyei S., Boampong F., Ocansey S. (2017). Symptomatic dry eye and its associated factors: a study of university undergraduate students in Ghana. *Eye & Contact Lens*.

[6] Sullivan D. A., Rocha E. M., Aragona P. (2017). TFOS DEWS II sex, gender, and hormones report. *The Ocular Surface*.

[7] Wickham L. A., Gao J., Toda I., Rocha E. M., Ono M., Sullivan D. A. (2000). Identification of androgen, estrogen and progesterone receptor mRNAs in the eye. *Acta OphthalmologicaScandinavica*.

[8] Rocha E. M., Wickham L. A., Silveira L. A. D. (2000). Identification of androgen receptor protein and 5*α*-reductase mRNA in human ocular tissues. *British Journal of Ophthalmology*.

[9] Wood R. L., Zhang J., Huang Z. M. (1999). Prolactin and prolactin receptors in the lacrimal gland. *Experimental Eye Research*.

[10] Mathers W. D., Stovall D., Lane J. A., Zimmerman M. B., Johnson S. (1998). Menopause and tear function: the influence of prolactin and sex hormones on human tear production. *Cornea*.

[11] Gibson E. J., Stapleton F., Wolffsohn J. S., Golebiowski B. (2017). Local synthesis of sex hormones: are there consequences for the ocular surface and dry eye?. *British Journal of Ophthalmology*.

[12] Saladin K. S. (1997). *Anatomy & Physiology: The Unity of Form and Function*.

[13] Gill-Sharma M. K. (2009). Prolactin and male fertility: the long and short feedback regulation. *International Journal of Endocrinology*.

[14] Rivarola M. A., Forest M. G., Migeon C. J. (1968). Testosterone, androstenedione and dehydroepiandrosterone in plasma during pregnancy and at delivery: concentration and protein binding. *Journal of Clinical Endocrinology and Metabolism*.

[15] Bammann B. L., Coulam C. B., Jiang N.-S. (1980). Total and free testosterone during pregnancy. *American Journal of Obstetric Gynecology*.

[16] Asiedu K., Kyei S., Mensah S. N., Ocansey S., Abu L. S., Kyere E. A. (2016). Ocular surface disease index (OSDI) versus the standard patient evaluation of eye dryness (SPEED): a study of a nonclinical sample. *Cornea*.

[17] Schiffman R. M., Christianson M. D., Jacobsen G., Hirsch J. D., Reis B. L. (2000). Reliability and validity of the ocular surface disease index. *Archives of Ophthalmology*.

[18] Bron A. J., Abelson M. B., Ousler G. (2007). Methodologies to diagnose and monitor dry eye disease: report of the diagnostic methodology subcommittee of the international dry eye workshop (2007). *Ocular Surface*.

[19] Tomlinson A., Bron A. J., Korb D. R. (2011). The international workshop on meibomian gland dysfunction: report of the diagnosis subcommittee. *Investigative Ophthalmology & Visual Science*.

[20] Bron A. J., Evans V. E., Smith J. A. (2003). Grading of corneal and conjunctival staining in the context of other dry eye tests. *Cornea*.

[21] Arita R., Minoura I., Morishige N. (2016). Development of definitive and reliable grading scales for meibomian gland dysfunction. *American Journal of Ophthalmology*.

[22] Asiedu K., Kyei S., Dzasimatu S. K., Morny E. K. (2018). Meibomian gland dysfunction in a youthful clinical sample in Ghana. *Optometry and Vision Science*.

[23] Field A. (2000). *Discopering Statistics Using SPSS*.

[24] Cook R. D., Weisberg S. (1982). *Residuals and Influence in Regression*.

[25] Myers R. H., Myers R. H. (1990). *Classical and Modern Regression with Applications*.

[26] Menard S. (1995). An introduction to logistic regression diagnostics. *Applied Logistic Regression Analysis*.

[27] Hu Y., Ding Y., Yang M., Xiang Z. (2018). Serum prolactin levels across pregnancy and the establishment of reference intervals. *Clinical Chemistry and Laboratory Medicine (CCLM)*.

[28] Kim S. Y., Sung Y. A., Ko K. S. (1993). Direct relationship between elevated free testosterone and insulin resistance in hyperprolactinemic women. *The Korean Journal of Internal Medicine*.

[29] Kyei S., Dzasimatu S. K., Asiedu K., Ayerakwah P. A. (2018). Association between dry eye symptoms and signs. *Journal of Current Ophthalmology*.

[30] Nichols K. K., Nichols J. J., Mitchell G. L. (2004). The lack of association between signs and symptoms in patients with dry eye disease. *Cornea*.

[31] Golebiowski B., Badarudin N., Eden J., You J., Hampel U., Stapleton F. (2017). Does endogenous serum oestrogen play a role in meibomian gland dysfunction in postmenopausal women with dry eye?. *British Journal of Ophthalmology*.

[32] Sullivan D. A., Sullivan B. D., Evans J. E. (2002). Androgen deficiency, Meibomian gland dysfunction, and evaporative dry eye. *Annals of the New York Academy of Sciences*.

[33] Krenzer K. L., Reza Dana M., Ullman M. D. (2000). Effect of androgen deficiency on the human meibomian gland and ocular surface. *The Journal of Clinical Endocrinology & Metabolism*.

[34] Azzarolo A. M., Bjerrum K., Maves C. A. (1995). Hypophysectomy-induced regression of female rat lacrimal glands: partial restoration and maintenance by dihydrotestosterone and prolactin. *Investigative Ophthalmology & Visual Science*.

